# Extramedullary hematopoiesis in a case of benign mixed mammary tumor in a female dog: cytological and histopathological assessment

**DOI:** 10.1186/1746-6148-6-45

**Published:** 2010-09-16

**Authors:** Fabrizio Grandi, Marcia M Colodel, Lidianne N Monteiro, João Rafael VP Leão, Noeme S Rocha

**Affiliations:** 1 Department of Clinical Veterinary Medicine, School of Veterinary Medicine and Animal Science - FMVZ, Univ. Estadual Paulista - UNESP, Botucatu, São Paulo, Brazil; 2 Department of Animal Reproduction and Veterinary Radiology - FMVZ, School of Veterinary Medicine and Animal Science - FMVZ, Univ. Estadual Paulista - UNESP, Botucatu, São Paulo, Brazil

## Abstract

**Backgroud:**

Extramedullary hematopoiesis (EMH) is defined as the presence of hematopoietic stem cells such as erythroid and myeloid lineage plus megakaryocytes in extramedullary sites like liver, spleen and lymph nodes and is usually associated with either bone marrow or hematological disorders. Mammary EMH is a rare condition either in human and veterinary medicine and can be associated with benign mixed mammary tumors, similarly to that described in this case.

**Case presentation:**

Hematopoietic stem cells were found in a benign mixed mammary tumor of a 7-year-old female mongrel dog that presents a nodule in the left inguinal mammary gland. The patient did not have any hematological abnormalities. Cytological evaluation demonstrated two distinct cell populations, composed of either epithelial or mesenchymal cells, sometimes associated with a fibrillar acidophilic matrix, apart from megakaryocytes, osteoclasts, metarubricytes, prorubricytes, rubricytes, rubriblasts, promyelocytes, myeloblasts. Histological examination confirmed the presence of an active hematopoietic bone marrow within the bone tissue of a benign mammary mixed tumor.

**Conclusions:**

EMH is a rare condition described in veterinary medicine that can be associated with mammary mixed tumors. It's detection can be associated with several neoplastic and non-neoplastic mammary lesions, i.e. osteosarcomas, mixed tumors and bone metaplasia.

## Background

Hematopoiesis in the adult animal is restricted to the marrow cavity of flat bones and long bones epiphysis [1]. Thus, extramedullary hematopoiesis (EMH) is defined as the presence of hematopoietic stem cells such as erythroid and myeloid lineage plus megakaryocytes in extramedullary sites and is usually associated with either bone marrow or hematological disorders [1-4]. Despite the fact that extramedullary hematopoieses can occur in any organ, it is more frequently seen in liver, spleen and lymph nodes [3-8].

Mammary EMH is a rare condition and it is generally associated with non-neoplastic hematopoietic masses in both woman and bitches. However, the presence of hematopoietic activity can also be seen as an incidental finding associated with mammary neoplasia [2,4,9-12].

Cytological examination has been used since the 60's to investigate mammary lesions in women, and it is nowadays largely accepted as a screening test for such lesions in veterinary medicine. It is a minimally invasive and low cost diagnostic method which allows differentiation between non-neoplastic and neoplastic mammary lesions. In addition, it can accurately predict the malignant potential of mammary tumors if performed by an experienced pathologist [13-18].

Based upon prevailing cytological characteristics, canine mammary tumors can be classified as epithelial, mesenchymal, or mixed type according to its origin [13,18].

Proliferation of epithelial cells from ducts and/or lobes, myoepithelial, and mesenchymal cells, in addition to cartilaginous, bone or myxoid fibrous tissue are sometimes seen in a variety of mammary neoplasm in dogs. These compound tumors are classified under the designation of mixed tumors [11,15,18-20], and represents up to 50% of the canine mammary tumors [19,21,22]. In humans, similar mammary lesions are uncommon, but are frequently seen in salivary glands [23,24].

Even though the tumor cytological examination may show considerable tissue heterogeneity [18], hematological stem cells are seldom observed. Hence, the objective of this case report was to describe the presence of hematopoietic stem cells in cytological samples of a spontaneous mixed benign mammary tumor in a bitch.

## Case presentation

A 7-year-old intact female non-pure breed dog weighing 8.0 kg was admitted at the School of Veterinary Medicine and Animal Science from the Univ. Estadual Paulista (UNESP), Botucatu, São Paulo, Brazil. On clinical examination it was noted a subcutaneous mass in the left inguinal mammary gland, measuring 0.7 × 0.9 cm, and with 24-week of clinical evolution. The lesion was also firm with a smooth surface, painless, and with no ulceration nor deep muscle adhesion.

Thoracic radiography, hematological and blood biochemistry analysis, and cytological evaluation of the lesion were performed in order to achieve a diagnosis. For cytological analysis, samples were obtained from 2 different areas using a fine needle (22 G1 1/4'', Injex^®^, São Paulo, Brazil) and a 10 ml syringe (Injex^®^, São Paulo, Brazil). The collected material was spread on five histological slides, and then fixed with methanol P.A. (Merck^®^, Darmstadt, Germany) and ethanol 95% (Merck^®^, Darmstadt, Germany). Three slides were stained with Giemsa and two with Papanicolaou stain. Samples were examined under light microscopy and classified according to Allen et al. [13] and Raskin and Meyer [18] standards for neoplastic mammary lesions.

After routine anesthetic procedures and local antisepsis, the animal was submitted to mastectomy. The tumor was collected, 10% formalin fixed, routinely processed and stained with hematoxylin and eosin (H&E). Then, the samples were analyzed under light microscopy.

## Results of hemogram, blood biochemistry and radiological examination

Hematological parameters and urea, creatinine, alkaline phosphatase, aspartate aminotransferase and alanine aminotransferase values were within reference interval considered normal for the specie (Table 1). Thorax radiographic examination did not find any detectable change.

**Table 1:** Hematological parameters and biochemical analysis of a female dog with benign mixed mammary tumor, showing extramedullary hematopoiesis

Parameters	Result	Reference value ***
**Erythrocyte **(x10^6^/μl)	6.13	5.50-8.50
**Hematocrit **(%)	40.00	37.00-55.00
**Hemoglobin **(g/dl)	15.00	12.00-18.00
**MCV **(pg)*	65.30	60.00-77.00
**MCHC **(%)**	36.00	32.00-36.00
**Total leukocyte **(x10^3^/μl)	11.20	6.00-17.00
**Segmented neutrophils **(x10^3^/μl)	6.80	3.00-11.50
**Rods neutrophils **(x10^3^/μl)	0.00	0.00-0.30
**Eosinophils **(x10^3^/μl)	0.60	0.10-1.25
**Lymphocytes **(x10^3^/μl)	4.60	1.00-4.80
**Monocytes **(x10^3^/μl)	0.20	0.15-1.35
**Platelets **(x10^3^/μl)	150.00	165.00-460.00
**Urea **(mg/dl)	30.30	21.40-59.92
**Creatinine **(mg/dl)	0.90	0.50-1.50
**Alkaline phosphatase **(UI/l)	87.20	20.00-156.00
**Aspartate aminotransferase **(UI/l)	11.80	6.20-13.00
**Alanine aminotransferase **(UI/l)	57.00	21.00-73.00
**Total proteins **(g/dl)	7.00	5.40-7.10
**Albumin **(g/dl)	2.70	2.60-3.30
**Globulin **(g/dl)	4.40	2.70-4.40

*MCV - Mean Corpuscular Volume

**MCHC - Mean Corpuscular Hemoglobin Concentration

***Jain [26].

## Results of cytological examination

**Figure 1:**
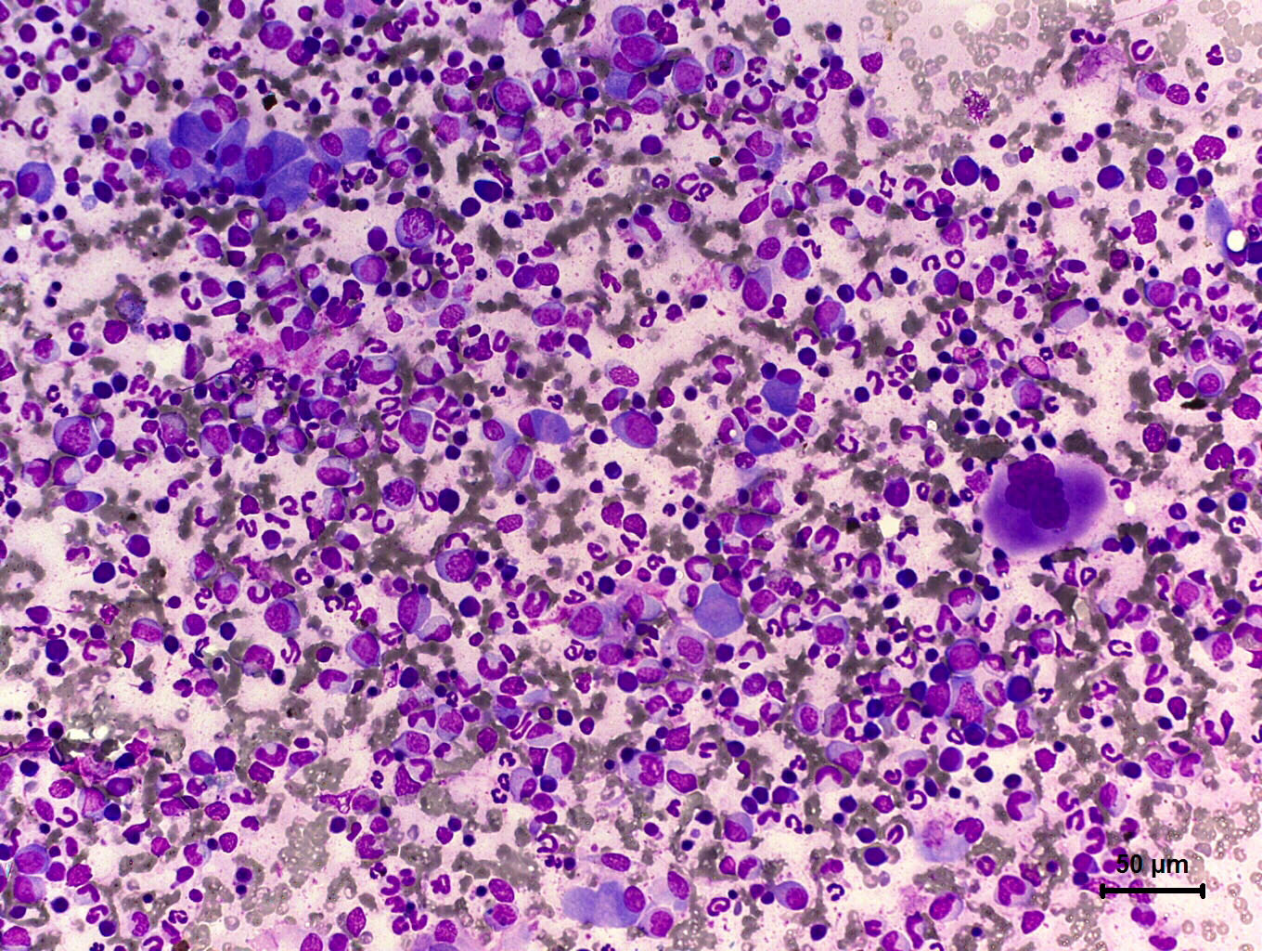
**Photomicrography of fine needle cytology from benign mixed mammary tumor in a non-purebred female dog**. Presence of erythroids and myeloids stem cells associated to a group of neoplastic epithelial cells. Giemsa, 40×.

**Figure 2:**
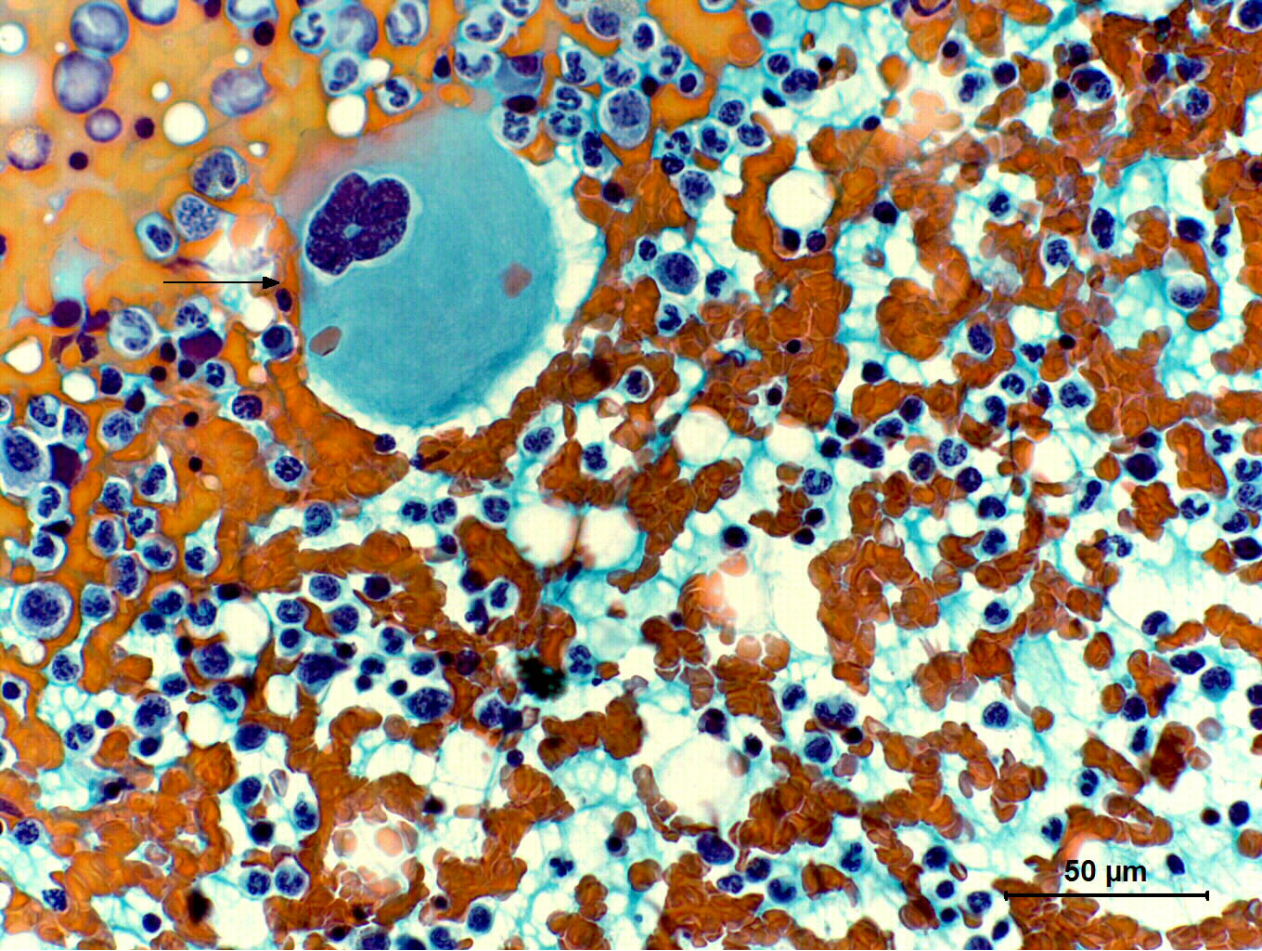
**Photomicrography of fine needle cytology from benign mixed mammary tumor in a non-purebred female dog**. Presence of a megakaryocyte (arrow). Papanicolaou, 40×.

Cytological evaluation (Figures 1 and 2) revealed high celullarity composed predominately by a high quantity of erythrocytes, moderate quantity of metarubricytes and discrete quantity of megakaryocytes, osteoclasts, rubricytes, promyelocytes, myeloblasts, prorubricytes and rubriblasts. Mitotic index was high, with typical mitosis. It was also seen other two cell populations: the first was composed by neoplastic epithelial cells arranged in tridimensional clusters with a moderate and indistinct basophilic cytoplasm with mild anisocytosis. Nuclei were oval and hyperchromatic, with mild anisokaryosis and indistinct nucleoli. No mitotic figures were observed; the second cell population was composed by scattered isolated fusiform cells, sometimes associated with fibrilar acidophilic matrix with moderate and mild basophilic cytoplasm. Nuclei were elongate and hyperchromatic. Nucleoli were indistinct.

## Results of histological analysis

**Figure 3:**
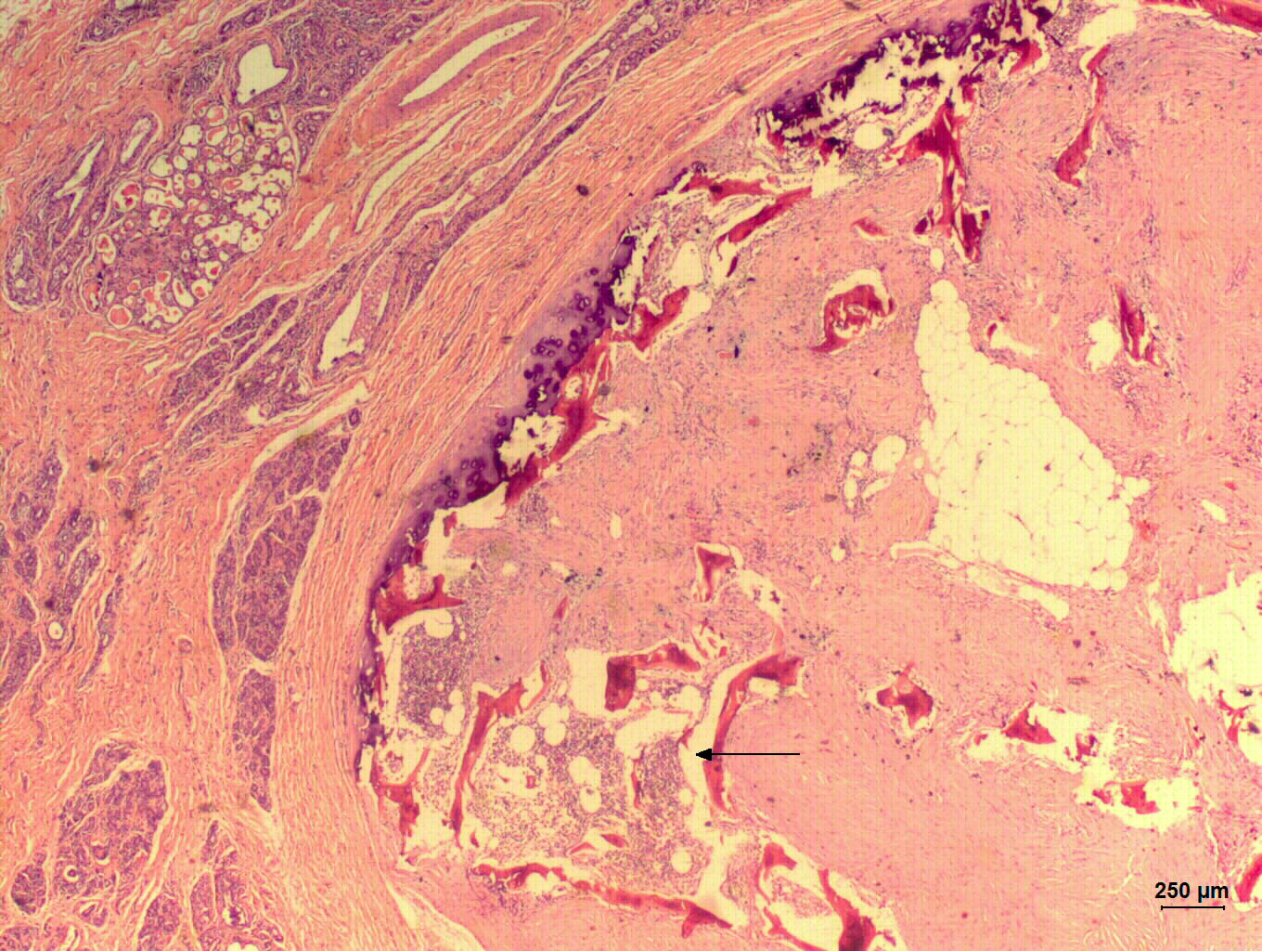
**Photomicrography of histological examination of a benign mixed mammary tumor in a non-purebred female dog**. Presence of hematopoietic active medulla (arrow) inside the neoplasia. Hematoxicilin and Eosin. 10×.

Histopathology revealed (Figure 3) an encapsulated and heterogeneous neoplasm composed by epithelial cells arranged in an acinar pattern presenting indistinct eosinophilic homogeneous cytoplasm, with clear vacuoles of various sizes and discrete anisocytosis. Nuclei were oval in shape with a loose chromatin pattern, a distinct small nucleolus and mild anisokaryosis. The myoepithelial component was composed by cells immersed in a myxoid matrix scattered between the acini, with distinct eosinophilic star-like cytoplasm. Nuclei were elongated and hyperchromatic with no distinct nucleoli. The mesenchymal component was composed by cartilage, bone and fat differentiation. The active bone marrow was composed by trilineage hematopoietic cells, i.e. myeloid, erytroid and megakaryocytic precursors. No mitosis was seen. We could also observe a high quantity of collagenous stroma.

## Discussion

Spontaneous mammary tumors in bitches are very similar to those in women, which makes it a good model for comparative studies [14,16] in terms of diagnostic, prognostic, behavioral, morphological, and biological approaches.

The mixed type, either benign or malignant, is one of the most common mammary gland tumor in bitches [19,21,22], unlike humans in which it is uncommon. However, the last ones have a high frequency of salivary gland neoplasia, denominated pleomorphic adenomas [23,24]. Benign mixed mammary tumors in dogs and pleomorphic adenomas in human salivary glands derive from exocrine glands that present similar architecture, and predominantly occur in females. Such tumors have morphological and molecular similarities which in turn suggests a similar pathogenic mechanism involved in malignant transformation [20,24].

The cytological examination usually shows that most of the pleomorphic adenomas present groups of epithelial, myoepithelial, and bone tissue cells, as well a chondromyxoid stroma [23]. Similarly, canine mixed mammary tumors may present epithelial, fibroblastic, cartilaginous, bone and rarely hematopoietic components. An acidophilic extracellular material, cell-associated or not, representing osteoid matrix could also be present. In addition, there may be various amounts of erytrocytes, foam cells, basophilic protein material and lipids on background [11,15,16,18-20]. Despite this, it is not common to find all these components in all samples [18] similarly to those described in this case. However, the representativeness of the lesion was guaranteed by its size and multiple sampling areas, which permits an accurate assessment of all neoplasm cytological components as confirmed later by histological analysis.

According to Allen et al. [13], during the cytological evaluation three or more architectural and/or nuclear malignancy criteria should be present in order to consider a diagnosis of a malignant tumor. In the present study, cytological evaluation of cell population on Giemsa and Papanicolaou stains did not show any of those criteria thus suggesting a benign tumor. The possibility of malignant transformation was minimal, since representativeness of the tumor mass was guaranteed by a multiple sampling technique.

The presence of one of the three lineages of hematopoietic cells out of the bone marrow is sufficient to characterize EMH [3,4]. In this case it was seen, apart from osteoclasts, some megakaryocytes, erythrocytes and myeloid lineage cells. These findings are similar to those described by Fernandes et al., [11] that observed erythrocytes and megakaryocytes in a benign mixed mammary tumor in a female dog and similar to the histological findings reported by Martinelli et al. [2], Brooks et al. [9], Setsu et al. [10], Cufer and Bracko [12]. Even before histological confirmation, these cells were observed in the cytological examination, which allowed a suggestive diagnosis of benign mixed mammary tumor with a hematopoietic active bone marrow in a very fast and precocious fashion, as demonstrated by Allen et al. [13], Hellmén and Lindgren [14], Zuccari et al. [15], Cassali et al. [16], Simon et al. [17]. However, cytologic evaluation alone did not permit us to exclude the diagnosis of a possible bone metaplasia or osteosarcoma.

EMH is generally seen in patients with bone marrow and hematological diseases such as chronic anemia, hematopoietic neoplasia, medular hyperplasia, suppurative bacterial infections, or cardiorespiratory conditions [1,3,4,8,25]. In such scenario, apart from the spleen, liver and lymphnodes, there can be microscopic hematopoietic areas in kidneys, lungs, gastrointestinal tract, adrenals, skin, heart, ovaries, epididymides, thymus, peritoneum, meninges and breast in humans [2-8], and choroid plexus and mammary gland in dogs [1,11].

Several hypothesis has been suggested to explain mammary EMH. Functional disruptions of bone marrow, e.g. drug therapy or myelofibrosis, stimulates circulating stem cells to find a favorable environment and differentiate into hematopoietic cells [4]. Focal lesions such as biopsies or surgeries are able to induce the production of growth factors or cytokines that activate hematopoiesis [6].

In this case, no biochemical or hematologic changes that could characterize a functional failure of bone marrow were detected, which could result in the production of chemical mediators capable of promoting favorable environment for stem cell implantation. The patient didn't suffer from any other disease, and was not under current drug therapy. No previous surgical manipulation of the mass was attempted. Thus, the pathogenesis of this case shall probably be related to intrinsic factors of the neoplastic process.

As far we know there are no reports in human medicine literature regarding the presence of erythrocytes, myeloids cells, and megakaryocytes in mixed or metaplastic breast tumors, and pleomorphic adenomas. In veterinary medicine there is only one report from Fernandes et al. [11] that reported the presence of two hematopoietic stem cell lineages in a cytological sample of a benign mixed mammary tumor in a dog. These authors did not describe the occurrence of medullar alterations in their study, making it an incidental finding just like in this case.

## Conclusions

Despite the existence of one previous report, the importance of those findings relies on the possible relationship with advanced medullar disorders. Moreover, mammary EMH is generally asymptomatic and clinically mimics a neoplasia.

Sampling multiple different sites of the same tumor mass increases its representativeness and possibility of an accurate diagnosis, since there is a heterogeneous tissue composition within the same tumor. Hence, the EMH cytological diagnosis is straightforward when hematopoietic cells are present. However it's important to emphasize that the simple fact of finding this cell types does not allow a final diagnosis, since other neoplastic and non-neoplastic condition, i.e. osteosarcomas and bone metaplasia, may present similarly. Despite all that, histopathological examination is always recommended.

## Authors' contributions

FG performed the analysis and interpretation of cytologic and histologic findings, photographed images and helped to write the manuscript. MMC participated in clinical research, sampling and helped to write the manuscript. LNM has contributed to the analysis and interpretation of cytology and histology and for the preparation of the manuscript. JRVPL conducted clinical research and was responsible for collecting samples. NSR is the supervisor responsible for the case report and the review of the manuscript. All authors read and approved the final manuscript.
